# Does Sound Timing Organization Matter? How Time Interval Influences the Perception of Closely Spaced Frequencies

**DOI:** 10.3390/brainsci16050439

**Published:** 2026-04-22

**Authors:** Krystsina Liaukovich, Olga Martynova

**Affiliations:** 1 Institute of Higher Nervous Activity and Neurophysiology of the Russian Academy of Sciences, 5A, Butlerova str., Moscow 117484, Russia; omartynova@ihna.ru; 2 Centre for Cognition and Decision Making, National Research University Higher School of Economics, 20, Myasnitskaya str., Moscow 101000, Russia

**Keywords:** auditory, perception, implicit, explicit, eMMN, MMR, P300, predictive coding

## Abstract

**Highlights:**

**What are the main findings?**

- Temporal predictability may enhance auditory frequency discrimination, but only under active listening and when discrimination is difficult; the local irregularity (five-tone bundles) yields higher hit rates and perceptual sensitivity (d’) compared to oddball or two-tone paradigms, accompanied by enhanced P300 amplitude.
- Participants may show a dissociation between objective performance and subjective awareness: they perform better in temporally predictable sequences yet do not consciously perceive them as easier, and substantial individual differences highlight that “one paradigm does not fit all.”

**What are the implications of the main findings?**

- Clinical and practical applications: Auditory assessments may incorporate temporally structured stimuli to avoid underestimating patients’ true discriminative capacity, and auditory displays (e.g., medical alarms) could be optimized using predictable temporal patterns to improve detection accuracy.
- Theoretical and methodological implications: The metacognitive dissociation observed suggests that subjective reports may not reliably reflect actual perceptual abilities—objective measures are essential; these findings support predictive coding frameworks, demonstrating that the brain leverages temporal regularity to enhance change detection even near threshold.

**Abstract:**

**Background/Objectives**: Temporal predictability may sharpen our ability to distinguish similar sounds, but whether this relies on attention is unclear. This study examined how temporal structure influences frequency discrimination. **Methods**: Thirty-six adults completed active (attend) and passive (ignore) listening tasks across three paradigms that varied in temporal structure: oddball (isolated deviants), two-tone frequency discrimination paradigm (pairs comparison), and local irregularity of the local/global paradigm (five-tone sequences, bundles). Stimuli varied in difficulty via small or large frequency deviations. Behavioral responses and subjective ratings were collected during active and passive listening. EEG was recorded to assess mismatch negativity (MMN) (either early MMN (eMMN) or mismatch response (MMR)) and P300 event-related potentials. **Results**: Under active listening, temporal predictability significantly improved performance, but only for difficult discriminations. The local-irregularity condition yielded higher hit rates and greater perceptual sensitivity (d’) than the other paradigms. This benefit was accompanied by enhanced P300, yet participants rated the conditions as equally difficult, indicating no metacognitive awareness. Under passive listening, predictability helped only for easy stimuli, marked by a larger MMR. No reliable change-detection response occurred for difficult sounds when attention was diverted. **Conclusions**: These findings suggest that the combination of temporal predictability and repeated standard presentation in the local irregularity paradigm can improve frequency discrimination under challenging, attended conditions, with some evidence for partial dissociation between objective performance and subjective awareness. However, substantial individual variability and cross-paradigm confounds caution against strong causal claims. These results are broadly consistent with predictive coding frameworks but require replication with counterbalanced designs and larger deviant trial counts.

## 1. Introduction

### 1.1. The Challenge of Auditory Perception in Time and Frequency

The auditory system operates in a complex acoustic environment, continuously processing sounds that vary not only in their spectral content but also in their temporal structure. A fundamental challenge in auditory perception is understanding how the brain integrates these two dimensions—frequency and time—to form coherent perceptual representations. While decades of research have established that listeners can discriminate subtle frequency differences under controlled conditions [1], far less is known about how temporal organization modulates this ability. Specifically, does presenting sounds in predictable temporal patterns enhance the perception of small frequency differences, and does this depend on whether the listener is actively attending or passively exposed?

Classic models of auditory scene analysis propose that the brain segments incoming acoustic information into perceptual streams based on frequency proximity and temporal regularity [2,3]. When sounds are presented at a fast rate, stimulus grouping may occur on the basis of temporal or spatial proximity or of some repetitive pattern of stimulation. Temporal unpredictability may disrupt stream formation and impair discrimination. However, the majority of frequency discrimination studies have employed either the oddball paradigm, where deviant sounds occur randomly among repeated standards [4,5], or the two-tone frequency discrimination paradigm, where listeners compare two sounds presented in succession [6]. Both designs introduce temporal uncertainty: in oddball paradigms, the deviant occurs at an unpredictable moment; in two-tone paradigms, the deviant pair occurs at an unpredictable moment. This uncertainty may obscure the listener’s true discriminative capacity, particularly when frequency differences are near threshold [4,6].

An alternative to temporally uncertain oddball and two-tone designs is the local/global paradigm, originally developed to study hierarchical predictive processing [7]. In its local variant, five-tone sequences (bundles) are presented, with deviants occurring only at the final, predictable position. In 1998, Sussman and colleagues [8] showed that when stimulus onset asynchrony (SOA) was shortened to 100 ms, which is the case in these bundles, the auditory system was able to group all five stimuli (SSSSD) as a single unit. This design creates strong temporal expectations: the listener knows when the potential deviant will occur. Previous research using this paradigm has focused primarily on ERP dissociations between local and global violations during passive listening [7,9], during falling asleep [10] or awakening [11], and during various unconscious conditions [12,13]. However, no study to date has systematically compared how the oddball paradigm, two-tone frequency discrimination paradigm, and local/global paradigm influence behavioral frequency discrimination performance across varying levels of task difficulty and attentional state.

### 1.2. Event-Related Potentials: eMMN, MMR, and P300

Event-related potentials (ERPs) provide a millisecond-precise window into the neural processes underlying auditory deviance detection and attention. Three components, i.e., eMMN, MMR, and P300, are particularly relevant to the present study.

The nature of mismatch negativity (MMN) remains an active area of discussion, with multiple hypotheses regarding its genesis (for reviews, see [14,15,16,17,18,19]).

From the classic point of view, MMN is a pre-attentive, frontocentral negative component typically peaking between 100 and 250 ms post-deviant onset, generated automatically regardless of attention [15,18], where MMN is regarded as the output of a cortical deviance detection system that compares incoming sensory input with a short-term memory trace of preceding regularities [18].

Contemporary research is heavily influenced by the predictive coding framework [20], which proposes that the brain continuously generates top-down predictions about sensory input; violations of these predictions generate prediction errors that drive perceptual updating. Temporal regularity allows the brain to predict when a stimulus will occur, sharpening sensory gain at predicted time points. In this case, standard stimuli establish a predictive model, and the deviant violates this prediction, generating a prediction error signal—MMN [20,21,22].

Dynamic Attending Theory [23,24] posits that endogenous oscillatory rhythms entrain to temporal regularities, creating temporal expectancies that enhance perceptual sensitivity at expected moments. Both frameworks predict that temporal predictability should facilitate discrimination, but they differ in whether this effect requires attention: predictive coding suggests some pre-attentive effects are possible, while dynamic attending emphasizes attention-dependent oscillatory entrainment.

However, based on the contribution of attention, MMN may be divided into several subcomponents [25].

Some studies have distinguished an early MMN (eMMN) component, peaking as early as 50–100 ms post-deviant onset with fronto-central topography, which is thought to reflect more basic, sensory-specific prediction errors processed in primary auditory cortex [18,25]. The eMMN is typically elicited by simple acoustic feature deviations (e.g., frequency, intensity) and is considered more automatic and less influenced by attentional state than the later MMN; however, it is debated by Sussman et al. (2014) [19] whether MMN is pre-attentive at all.

MMR is a separate negative component (150–300 ms) with a more frontal distribution that reflects involuntary attention capture [10,25]. Unlike eMMN, which can be elicited pre-attentively, MMR is typically observed when deviants are salient enough to break through ignored instructions and automatically orient attention towards the sound. MMR is similar to the N2b component, which is also characterized by frontal distribution [26,27]; therefore, it is too difficult to differentiate them [19]. MMR is characterized by the activation of not only the primary auditory cortex but also the activity of frontal areas [18,28,29,30].

P300 (or P3) is a positive component typically maximal over parietal electrodes, peaking between 250 and 600 ms post-stimulus depending on task difficulty [31]. P300 is thought to reflect attention-driven context updating when a deviant stimulus is detected and categorized. Its amplitude is proportional to the attentional resources allocated to the deviant, and its latency indexes stimulus evaluation time [31]. P300 is generally observed only under active attention conditions, making it a reliable marker of explicit deviance detection and categorization. Within the predictive coding framework, P300 may reflect the updating of higher-order predictions following a detected prediction error [20].

In the present study, we use these ERP components to dissociate pre-attentive (eMMN), attention-capturing (MMR), and attention-dependent (P300) processes across paradigms and listening states.

### 1.3. Psychophysical Background on Frequency Discrimination

Classic psychophysical studies have established that frequency discrimination thresholds (difference limens) for pure tones typically range from 1 to 3 Hz at low frequencies (≤1000 Hz) to 10–20 Hz at higher frequencies, with performance improving with longer stimulus durations and shorter inter-stimulus intervals [1,32]. The just-noticeable difference for harmonic complex tones is similar to that for pure tones with matching fundamental frequencies [33]. Critically, temporal factors, including SOA, inter-stimulus interval (ISI), and temporal regularity, have been shown to modulate frequency discrimination thresholds [18]. However, these classic studies have primarily used forced-choice paradigms with explicit comparisons, leaving open the question of how temporal structure influences the detection of infrequent deviants in more ecologically valid streaming contexts.

Although research investigating the mechanisms underlying the discrimination of sounds close in frequency has been ongoing for more than forty years, the question of whether the organization of stimulus presentation influences the efficiency of their perception remains open. In our 2022 study [9], we presented single stimuli (oddball paradigm) and then included the same stimuli organized into bundles of five sounds (local/global paradigm). In response to local irregularity, we recorded the P3a event-related potential component, which reflects (un)conscious processing of the difference between stimuli [31]. Therefore, in that previous study, we hypothesized that, due to training on single stimuli, a learning process may have occurred into more complex patterns. However, we decided to continue the research to clarify whether this hypothesis is correct or whether it was not so much the training but rather the organization of stimuli that was important for successful discrimination.

### 1.4. The Present Study: Aims, Questions, and Hypotheses

The present study addressed the gap in systematic cross-paradigm comparison. We asked three primary questions:

First, does temporal grouping (local irregularity of the local/global paradigm—bundles of five sounds) improve frequency discrimination compared to isolated oddball or paired presentations, even when frequency differences are subtle?

Second, are these effects mediated by attention? That is, do they emerge only under active listening, or can they be observed pre-attentively, as indexed by eMMN?

Third, do participants have metacognitive awareness of which paradigm facilitates their performance?

To answer these questions, we recorded behavioral responses (hit rate, reaction time, and d’), subjective ratings, and ERPs (eMMN, MMR, and P300) from thirty-six adults who completed all three paradigms under both passive and active listening states. Stimuli were harmonic tones differing from a standard tone (480–960–1440 Hz) by either small (485–970–1455 Hz; difficult condition) or large (520–1040–1560 Hz; easy condition) frequency deviations.

### 1.5. Operational Definitions

Before proceeding, we define key terms operationally as used in this study.

Implicit processing refers to effects that are observed in objective performance (e.g., higher hit rates or d’) without corresponding explicit awareness as measured by post-block subjective ratings. This operational definition does not imply that the processing is unconscious in the strong sense (e.g., subliminal perception), only that participants did not explicitly report awareness of the paradigm differences that benefited their performance.

Outside awareness/Metacognitive dissociation is defined as a statistically significant difference between objective performance measures (hit rate, d’) and subjective ratings of perceived ease or efficiency. This is a relative dissociation (performance differs but ratings do not) rather than an absolute one (performance above chance with ratings at chance).

Pre-attentive describes processing that occurs when participants are instructed to ignore auditory stimuli and engage in a visual distractor task (watching a muted nature documentary). We do not claim that such processing is entirely automatic or occurs without any neural resource allocation; rather, we use ‘pre-attentive’ to distinguish the passive listening state from the active state where participants explicitly attended to sounds and made behavioral responses.

## 2. Results

### 2.1. Subjective Assessment of Stimuli in the Passive State

Following the passive listening blocks, participants reported their primary focus of attention. The majority of participants reported watching the video (75% in the difficult condition; 63.9% in the easy condition) or mind-wandering (16.7% in the difficult condition; 25% in the easy condition). Only one participant (2.8%) reported actively listening to the sounds during the difficult condition, and no participants reported active listening during the easy condition. A small proportion of participants reported engaging in other activities (5.5% in the difficult condition; 11.1% in the easy condition), which mostly consisted of a combination of watching the video and mind-wandering. These self-reports confirm that the passive condition successfully minimized direct attention to the auditory stimuli.

**Figure A1:**
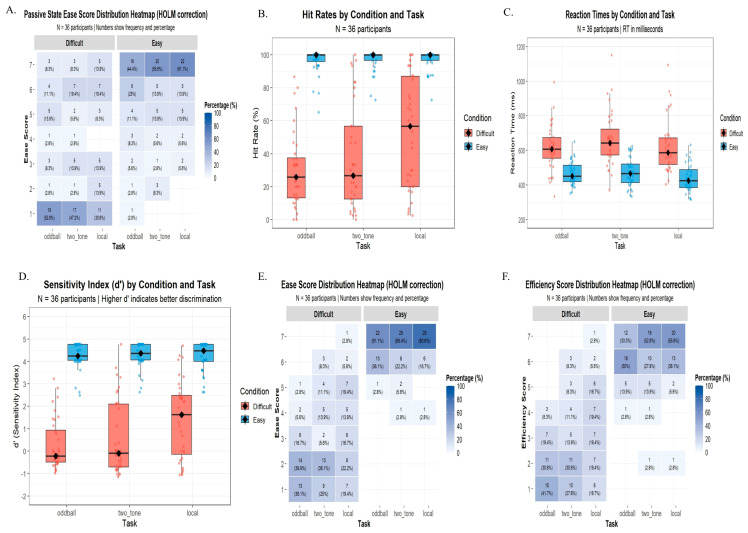
Behavioral results and subjective rating of ease to differentiate sounds across paradigms and difficulty levels (the passive and active states) in 36 participants. (**A**) Perceived ease score distribution heatmap in the passive state. (**B**) Hit rates by condition and task in the active state. (**C**) Reaction time by condition and task in the active state. (**D**) Sensitivity index by condition and task in the active state. (**E**) Perceived ease score distribution in the active state. (**F**) Efficiency score distribution heatmap in the active state.

A Friedman test revealed significant main effect of paradigm on subjective assessment of the ease of perception in the easy condition (χ^2^(N = 36, df = 2) = 10.8, *p* = 0.005) but not in the difficult condition (χ^2^(N = 36, df = 2) = 3.97, *p* = 0.137) (Table 1, Table A1 and Table A2, Figure A1A).

In the difficult condition, no significant differences were found between paradigms (all *p* > 0.1), suggesting that participants did not consciously perceive one paradigm as easier than another when discrimination was challenging under passive listening.

In the easy condition, the local irregularity was assessed as easier to perceive than the oddball paradigm. This may indicate that even in passive listening, temporal grouping may enhance the perceptual salience of large frequency differences.

Participants rated easy-to-differentiate sounds as significantly easier to distinguish than difficult-to-differentiate sounds across all paradigms, confirming that perceptual difficulty was subjectively experienced as intended (all *p* < 0.001).

### 2.2. eMMN/MMR in the Passive State

#### 2.2.1. Within Difference in eMMN/MMR in Two Conditions

Time windows were identified based on cluster-based permutation tests and reflect the amplitude range in which the deviant-standard difference was significant.

**Figure 1:**
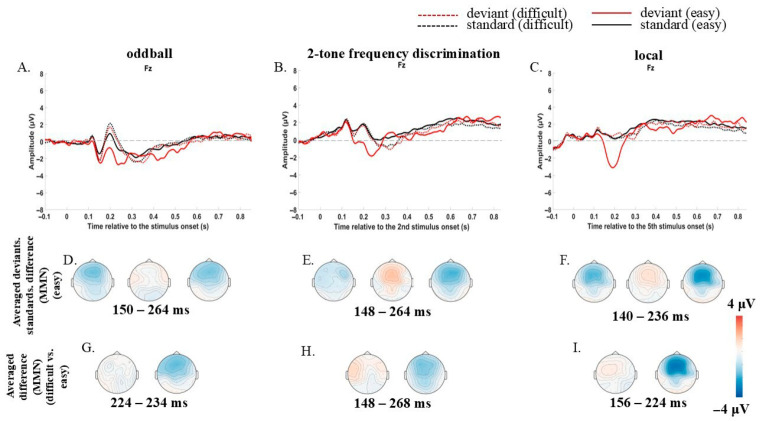
MMR in the passive state. (**A**–**C**) ERPs at Fz: standards (red) vs. deviants (black); solid = easy, dashed = difficult conditions. (**D**–**F**) Topography of deviant-standard difference (easy condition only). (**G**–**I**) MMR topography: easy (right) vs. difficult (left). Light grey dashed line serves as a baseline. Significant time windows (cluster-based permutation, *p* < 0.025) are indicated.

There was a significant difference in the mean amplitude of ERPs in the easy condition, not the difficult one. MMR in the oddball paradigm was registered in the 150–264 ms window (*p* = 0.002). MMR in the two-tone frequency paradigm was registered in the 148–264 ms window (*p* = 0.002). MMR in local irregularity was registered in the 140–236 ms window (*p* = 0.002). (Figure 1A–F).

Two conditions differed in MMR, i.e., it was registered for the easy condition, not for the difficult one (*p* = 0.018, *p* = 0.004, and *p* = 0.002 for oddball, two-tone frequency discrimination paradigms and local irregularity, respectively) (Figure 1G–I).

#### 2.2.2. Difference in MMN Between Paradigms in Two Conditions

**Figure 2:**
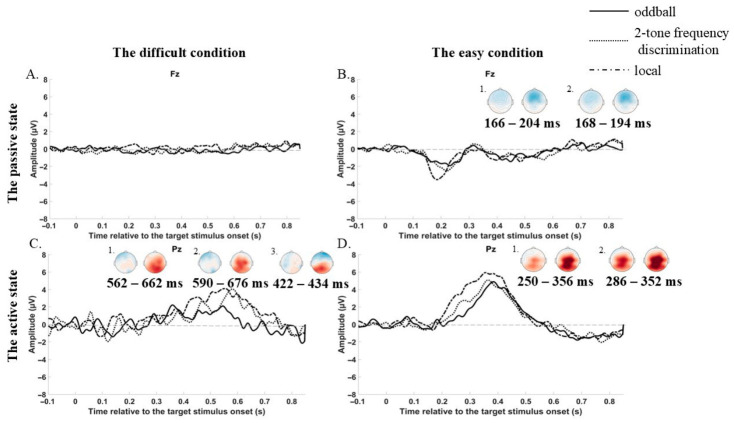
Cross-paradigm eMMN/MMR and P300 comparisons. (**A**,**B**) MMR difference waves at Fz; topography of paradigm differences. (**B1**) The difference between the oddball paradigm and the local irregularity (right). (**B2**) The difference between the two-tone frequency paradigm (left) and the local irregularity (right). (**C**,**D**) P300 difference waves at Pz; topography of paradigm differences. (**C1**) The oddball paradigm (left) and two-tone frequency discrimination paradigm (right). (**C2**) The oddball paradigm (left) and local irregularity (right). (**C3**) The two-tone frequency discrimination paradigm (left) and local irregularity (right). (**D1**) The oddball paradigm (left) and local irregularity (right). (**D2**) The two-tone frequency discrimination paradigm (**left**) and local irregularity (**right**). Light grey dashed line serves as a baseline. Significant time windows (cluster-based permutation, *p* < 0.025) are indicated.

No significant differences were observed among the paradigms in the difficult condition (Figure 2A).

In the easy condition, MMR amplitude was significantly larger in local irregularity compared to both the oddball (166–204 ms, *p* = 0.006) and the two-tone frequency discrimination (168–194 ms, *p* = 0.016) paradigms. No significant differences were observed between oddball and two-tone frequency discrimination paradigms (Figure 2B).

### 2.3. Behavioral Results: The Subjective Assessment in the Active State

Behavioral performance and subjective ratings were analyzed across three presentation paradigms (oddball—one sound, two-tone frequency discrimination—pair of sounds, and local irregularity—a bundle (five sounds)) under two discrimination difficulty conditions (difficult vs. easy). Results are summarized in Table 2, Table 3, Table 4 and Table 5 and described below.

#### 2.3.1. Effects of Discrimination Difficulty

A strong main effect of difficulty was observed across all measures (*p* < 0.001, Table 2, Table A1 and Table A2, Figure A1B–F). In the easy condition, hit rates approached the ceiling, d’ values were high, reaction times were fast, and subjective ratings indicated high perceived ease and efficiency. In contrast, the difficult condition yielded significantly lower hit rates, reduced d’, slower RTs, and poorer subjective ratings.

#### 2.3.2. Behavioral Results: The Difficult Condition

When discrimination was difficult, paradigm type significantly influenced hit rate (χ^2^(N = 36, df = 2) = 16.00, *p* < 0.001) and d’ (χ^2^(N = 36, df = 2) = 8.85, *p* = 0.012) but not RT (χ^2^(N = 33, df = 2) = 1.7, *p* = 0.428) (see Appendix A Table A1 and Table A2, Figure A1B–D).

The local irregularity yielded the highest hit rate, the two-tone frequency discrimination paradigm was intermediate, and the oddball paradigm was the most difficult for performance.

For perceptual sensitivity, d’ was significantly higher for the local irregularity compared to both the oddball and two-tone frequency discrimination paradigms. However, the oddball and two-tone frequency paradigms did not differ from each other in the sensitivity index.

Beyond the group-level effects, we examined individual patterns. Using d’ as the primary metric, we classified each participant’s best-performing paradigm in the difficult condition. Results showed: twenty-one participants (58.3%) performed best on the local irregularity, nine (25.0%) on the two-tone paradigm, and six (16.7%) on the oddball paradigm. Notably, eight participants (22.2%) showed ceiling-level performance (d’ > 1) across all paradigms, suggesting that for these individuals, frequency discrimination was effortless regardless of temporal structure. Conversely, three participants (8.3%) failed to detect any deviants in at least one paradigm (two in oddball, one in two-tone), indicating that temporal uncertainty rendered them unable to perform the task at all. These individual differences were not explained by age, sex, or self-reported musical experience (all *p* > 0.20), suggesting other factors (e.g., working memory capacity, inborn music perception, temporal processing ability) as potential moderators.

Paradigms did not differ on RT. It is also important to note that three participants failed to detect any deviants at all (two in the oddball paradigm and one in the two-tone frequency discrimination paradigm) (see Appendix A, Table A1 and Table A2, Figure A1B–D), underscoring that for some individuals, temporal uncertainty left them unable to detect any deviants.

#### 2.3.3. Behavioral Results: The Easy Condition

No meaningful differences between paradigms were observed in the easy condition for hit rate (χ^2^(N = 36, df = 2) = 3.08, *p* = 0.214) or d’ (χ^2^(N = 36, df = 2) = 0.76, *p* = 0.685), indicating a ceiling effect. Significant differences in RT were noted (χ^2^(N = 36, df = 2) = 10.72, *p* = 0.005), with local bundles yielding the fastest responses (see Appendix A Table A1 and Table A2, Figure A1B–D).

### 2.4. Event-Related Potentials in the Active State

#### 2.4.1. Behavioral Performance

Because the analysis window was limited to 850 ms after target stimulus onset, only trials with motor responses occurring within this interval were included. Data was therefore reanalyzed using only the left epoch. Data of five participants, whose RT exceeded 850 ms or who did not respond at all in some tasks, were omitted from the final analysis in all conditions (N = 5) (Table 3, Table A1 and Table A2).

A Friedman test revealed a significant main effect of the paradigm on hit rate in the difficult condition (χ^2^(N = 36, df = 2) = 15.38, *p* < 0.001). Hit rate was significantly higher for the local irregularity compared to both oddball and 2-tone frequency discrimination paradigms. Oddball and two-tone frequency discrimination did not differ from each other in hit rate.

No significant main effect of the paradigm on hit rate in the easy condition was found (χ^2^(N = 36, df = 2) = 0.28, *p* = 0.871).

Interestingly, a Friedman test revealed a significant main effect of the paradigm on RT in the difficult condition (χ^2^(N = 31, df = 2) = 10.06, *p* = 0.007). RT was significantly higher both for the local irregularity and the two-tone frequency discrimination paradigm in comparison to the oddball paradigm. The difference between local irregularity and the two-tone frequency discrimination paradigm was not found.

A significant main effect of the paradigm on RT in the easy condition (χ^2^(N = 36, df = 2) = 7.03, *p* = 0.03). RT was significantly higher for the local paradigm compared to the two-tone frequency discrimination paradigm and tended to differ from the oddball paradigm. The difference between oddball and two-tone frequency paradigms was not found.

#### 2.4.2. ERPs in the Active State

**Figure 3:**
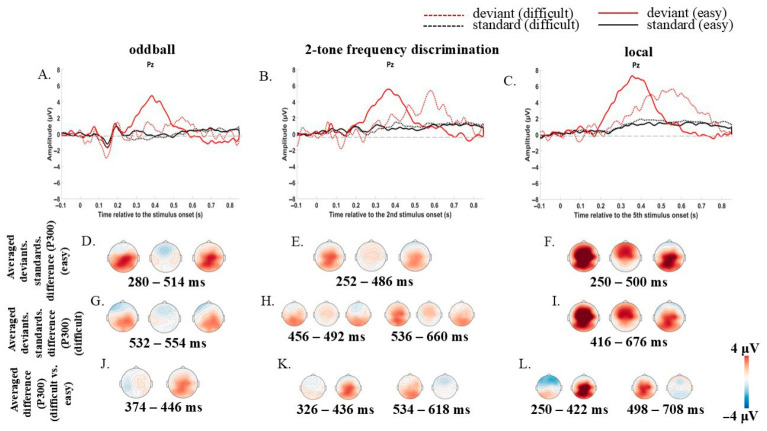
P300 in active condition. (**A**–**C**) ERPs at Pz: standards (red) vs. deviants (black); solid = easy, dashed = difficult. (**D**–**F**) P300 topography (easy condition). (**G**–**I**) P300 topography (difficult condition). (**J**–**L**) P300 comparison: easy vs. difficult. Light grey dashed line serves as a baseline. Significant time windows (cluster-based permutation, *p* < 0.025) indicated.

The cluster permutation test revealed significant P300 in both easy and difficult conditions (Figure 3).

In the easy condition, P300 in the oddball paradigm was registered in the 280–514 ms window (*p* = 0.002), in the two-tone frequency discrimination paradigm in the 252–486 ms window (*p* = 0.002), and in local irregularity in the 250–500 ms window (*p* = 0.002). (Figure 3D–F).

In the difficult condition P300 was also registered but with increased latency, i.e., in the oddball paradigm it was registered in the 532–554 ms window (*p* = 0.025), in the two-tone frequency discrimination paradigm in the 456–492 ms (*p* = 0.008) and 536–660 ms windows (*p* = 0.002) and in local irregularity in the 416–676 ms window (*p* = 0.002). (Figure 3G–I).

However, two conditions differed in P300. P300 was larger for the easy condition in all three paradigms in the early windows (in the oddball paradigm (374–446 ms, *p* = 0.01); in the two-tone frequency discrimination paradigm (326–436 ms, *p* = 0.004) and in local irregularity (250–422 ms, *p* = 0.003)) and it was larger for the difficult condition in the two-tone frequency discrimination paradigm (534–618 ms, *p* = 0.004) and local irregularity (498–708 ms, *p* = 0.003) in later windows since its latency enlarged (Figure 3J–L).

#### 2.4.3. Difference in P300 Between Paradigms in Two Conditions

The cluster permutation test indicated that in the difficult condition, P300 was larger in the local irregularity in comparison to the oddball paradigm (in the 590–676 ms window, *p* = 0.02) and to the two-tone frequency discrimination paradigm (in the 422–434 ms window, *p* = 0.025) (Figure 2C). And there was a difference between the oddball paradigm and 2-tone frequency discrimination paradigm, i.e., P300 amplitude was larger in the 2-tone frequency discrimination paradigm (in the 562–662 ms window, *p* = 0.002).

In the easy condition, P300 was larger in the local irregularity in comparison to the oddball paradigm (in the 250–356 ms window, *p* = 0.006) and to the two-tone frequency discrimination paradigm (in the 286–352 ms window, *p* = 0.022) (Figure 2D).

### 2.5. Subjective Assessment in Both Conditions in the Active State

#### 2.5.1. Subjective Ratings of Perceptual Ease in Both Conditions

##### Perceived Ease Across Paradigms in Both Conditions

Subjective ratings of how easy it was to discriminate sounds varied across paradigms, but this awareness depended on task difficulty (Table 4, Table A1 and Table A2, Figure A1E).

In the difficult condition, a Friedman test revealed a significant main effect of the paradigm on perceived ease (χ^2^(N = 36, df = 2) = 16.58, *p* < 0.001). The participants rated the oddball paradigm as more difficult to differentiate than the two-tone frequency discrimination paradigm and local irregularity. But no such difference was found between the two-tone frequency discrimination paradigms and local irregularity.

In the easy condition, a significant main effect of the paradigm was also observed (χ^2^(N = 36, df = 2) = 7.17, *p* = 0.028) but did not reach significance after post hoc. Here, the participants had a tendency to rate the local paradigm as significantly easier to differentiate than the 2-tone frequency discrimination paradigm, but not the oddball paradigm.

##### Subjective Ranging of the Paradigms Based on Perceived Ease

When asked to rank paradigms from easiest to most difficult at the end of the active blocks, participants showed no clear consensus in the difficult condition (Table 5), consistent with the absence of strong subjective differentiation during task performance.

Crucially, subjective ratings of ease did not fully align with objective performance (d’). Although the local paradigm yielded significantly higher hit rates and d’ than the two-tone paradigm in the difficult condition, participants did not rate it as significantly easier. These findings suggest partial and inconsistent metacognitive awareness. While participants rated the local irregularity as subjectively easier than the oddball paradigm, they did not rate it as significantly easier than the two-tone paradigm, despite objectively performing better on the local irregularity. The correlations between subjective efficiency ratings and objective d’ were significant for all paradigms, indicating that participants had some insight into their performance. However, the correlation for the oddball paradigm was weakest and fell just short of significance, suggesting that metacognitive accuracy may be reduced under conditions of high temporal uncertainty. Also, it is important to note that although bundles and pairs were rated as significantly easier than the oddball paradigm, absolute ratings remained low (<2 on a 7-point scale), reflecting the overall difficulty of the task. Participants knew the task was hard, but they did not know when they were performing better.

#### 2.5.2. Subjective Ratings of Performance Efficiency and Metacognitive Awareness

##### Perceived Efficiency Across Paradigms in Both Conditions

Beyond ratings of perceptual ease, we also asked participants to evaluate how efficiently they performed the task—that is, how few mistakes they believed they made (Table 5, Table A1 and Table A2, Figure A1F).

In the difficult condition, a Friedman test revealed a significant main effect of the paradigm on perceived efficiency (χ^2^(N = 36, df = 2) = 21, *p* < 0.001). Post hoc showed that participants believed they made the fewest mistakes in the local irregularity, the two-tone frequency discrimination paradigm was intermediate, and they made the most mistakes in the oddball paradigm.

In the easy condition, no significant main effect of the paradigm on perceived efficiency was observed (χ^2^(N = 36, df = 2) = 5.71, *p* < 0.058), consistent with the ceiling effects observed in objective performance.

##### Relationship Between Subjective Awareness and Objective Performance

To examine how well participants’ subjective judgments aligned with their actual performance, we calculated Spearman correlations between subjective ratings (perceived efficiency) and objective measures (hit rate and d’) for each paradigm and difficulty level (Table 6).

In the difficult condition, subjective efficiency ratings correlated significantly with hit rate for all three paradigms. In the easy condition, significant correlations were also observed for all three paradigms. These results indicate that participants had good metacognitive insight into how many deviants they correctly detected, regardless of task difficulty or paradigm.

For d’, which accounts for both hits and false alarms, a more nuanced pattern emerged. In the difficult condition, significant correlations were observed for the two-tone paradigm and local irregularity, but the correlation for the oddball paradigm fell just short of significance. In the easy condition, the local irregularity and the two-tone paradigm showed a significant correlation and the oddball paradigm was non-significant.

Together, these findings reveal a dissociation between metacognitive access to hit rate versus perceptual sensitivity. While participants demonstrated awareness of how many deviants they detected, their awareness of overall perceptual sensitivity, which incorporates false alarms and thus reflects discriminability independent of response bias, was weaker and more variable across paradigms. Critically, metacognitive accuracy for d’ was lowest in the oddball paradigm, the condition with the greatest temporal uncertainty. This pattern suggests that temporal unpredictability may impair metacognitive monitoring of perceptual sensitivity, potentially because unpredictable deviant timing makes it more difficult for participants to form a stable internal metric of their own performance accuracy.

## 3. Discussion

The present study investigated whether the temporal organization of sound sequences influences the perception of closely spaced frequencies under active and passive listening states. Our results demonstrate that temporal grouping robustly enhances auditory discrimination, but this benefit is conditional. It emerges only when discrimination is effortful and listening is active, and it operates, at least in part, outside of conscious awareness. Under a passive state, the benefit of temporal structure was evident only for easily discriminable sounds at the neurophysiological level.

### 3.1. Temporal Predictability Reduces Perceptual Uncertainty

The key finding was that, in the difficult condition, the local irregularity consistently outperformed both the oddball and two-tone frequency discrimination paradigms across all behavioral measures: hit rate and perceptual sensitivity. This advantage cannot be attributed to stimulus factors alone, as the physical frequency differences were identical across paradigms. Rather, it may reflect a cognitive benefit of temporal predictability.

It is important to acknowledge, however, that our paradigm comparison does not isolate temporal predictability as the sole causal factor. The local irregularity condition combines temporal predictability (the deviant always occurs at the fifth position) with a larger number of preceding standards (four) and a shorter within-bundle ISI (100 ms), compared to the oddball paradigm, which has a variable number of preceding standards and a 1000 ms SOA. Future studies using parametrically varied ISI and standard repetition counts are needed to dissociate these factors. Nevertheless, the consistent advantage of the local irregularity across multiple measures, coupled with the ERP findings of enhanced P300 (even compared to the two-tone paradigm, which also uses a 500 ms ISI), suggests that temporal predictability contributes meaningfully beyond mere standard repetition.

As was mentioned above, in the local irregularity paradigm, the deviant always occurs at the fifth position. This may allow listeners to allocate attention precisely in time, creating a temporal window of expectation that increases sensory gain and optimizes decision criteria [8]. In contrast, the oddball paradigm requires continuous monitoring; the listener must maintain a sensory memory trace of the standard and compare each incoming sound to that trace, with no temporal cue to guide attention [8]. The two-tone frequency discrimination paradigm reduces some uncertainty by presenting the two to-be-compared tones in close succession, but the onset of the pair itself remains unpredictable. Thus, the local irregularity uniquely combines local temporal predictability (knowing when the deviant will occur) with local spectral regularity (four identical standards preceding the deviant) [8]. These findings suggest that rapid temporal grouping facilitates frequency discrimination, likely by promoting auditory pattern integration and direct comparison within sensory memory windows [18].

This interpretation is consistent with predictive coding frameworks [20,34], which posit that the brain continuously generates predictions about upcoming sensory input. When predictions are violated (i.e., a deviant occurs), a prediction error signal is generated. The more precise the prediction—in both what and when—the larger and earlier the error signal.

The absence of reaction time differences between paradigms in the difficult condition is also informative. Although participants detected more deviants in the local irregularity, they did not respond faster. This may suggest that the benefit of temporal predictability operates primarily on perceptual sensitivity and detection accuracy rather than on motor preparation or response execution. In other words, temporal structure helps listeners notice the deviant, but once noticed, the response unfolds at a similar speed across paradigms.

Beyond the factors discussed above, the three paradigms differ along several additional dimensions that warrant consideration.

First, the paradigms differ in memory load. The oddball paradigm requires maintaining a single standard representation over time; the two-tone paradigm requires comparing two temporally proximal sounds; and the local irregularity requires maintaining a five-tone sequence representation.

Second, they differ in decision demands. In the oddball paradigm, participants must detect a single deviant among standards; in the two-tone paradigm, they must detect a deviant pair; and in the local irregularity paradigm, they must detect a deviant only in the fifth position.

Third, they differ in the number of comparisons per trial. The local irregularity offers four standards before each deviant, potentially creating a stronger sensory memory trace.

These differences mean that the effects observed between paradigms cannot be uniquely attributed to temporal predictability alone. We therefore interpret our results as demonstrating that the combination of factors in the local irregularity yields performance benefits, while acknowledging that isolating the unique contribution of temporal predictability would require additional control conditions.

### 3.2. MMN Is Modulated by Discriminability and Temporal Structure

The absence of a reliable change-detection response for difficult-to-discriminate sounds in the passive condition may reflect several methodological factors. First, the 5–15 Hz deviation may have approached the perceptual difference limen for some participants, particularly without explicit task demands. Second, the temporal jitter in SOA (±50 ms) may have attenuated MMN/MMR amplitude, and with only 30 deviant trials per condition, we may have been underpowered to detect small effects. Third, the harmonic complexity of the stimuli could have reduced the salience of frequency deviations. Thus, while our data may suggest that pre-attentive mechanisms may be insufficient for near-threshold frequency differences [4] under these specific experimental parameters, future studies with larger deviations, fixed SOA, and higher trial counts are needed to determine whether temporal predictability can facilitate pre-attentive discrimination of subtle frequency changes.

For easy-to-discriminate sounds, however, a clear change-detection response was present in all paradigms, and critically, the response for the local irregularity was significantly larger than for both the oddball and two-tone frequency discrimination paradigms. The frontocentral distribution and latency (140–200 ms) of this effect are more characteristic of MMR than eMMN. This suggests an involuntary attentional capture mechanism [10,25]. As was mentioned in Section 3.1, repeated presentation of four identical standards in the local irregularity creates a highly stable perceptual representation; the fifth sound is compared against this well-formed trace [8,35]. When that sound deviates, it is more likely to break through and capture attention, even when the listener is engaged elsewhere. In the oddball paradigm, standards are also repeated, but they are interleaved with deviants at longer, jittered SOAs, e.g., 1 s, which may weaken trace consolidation.

It is also worth noting that temporal jitter (±50 ms) was applied across all three paradigms specifically to equate global temporal unpredictability, thereby isolating the effect of local temporal structure (i.e., within-bundle predictability in the local irregularity paradigm) from general temporal expectations about when a sound might occur. While classical MMN studies often use fixed SOAs, recent evidence suggests that moderate jitter does not abolish MMN but may reduce its amplitude slightly [36]. Critically, because jitter was applied equally across all paradigms, any differences observed between paradigms cannot be attributed to this factor.

Moreover, the operational definition of ‘standard’ differs fundamentally across the three paradigms, which complicates direct comparisons of eMMN/MMR amplitudes. In the oddball paradigm, the standard is defined by global probability (80% occurrence) and establishes a memory trace through repeated presentation of identical sounds with variable inter-stimulus intervals. In the two-tone paradigm, the ‘standard’ refers to identical tone pairs, requiring the listener to compare two successive sounds and detect when the second tone deviates; this involves a different predictive model—specifically, a local comparison rather than a global probabilistic expectation. In the local irregularity paradigm, the standard is defined by sequence-based expectation, where four identical tones establish a strong temporal and spectral prediction for the fifth tone. These differences imply that the ‘prediction error’ signaled by eMMN/MMR reflects different computational processes across paradigms: global probability violation (oddball), local comparison violation (two-tone), and temporally precise sequence violation (local irregularity). The larger MMR we observed for the local irregularity in the easy passive condition may therefore reflect not only stronger sensory memory traces (due to four consecutive standards) but also the engagement of a different predictive mechanism.

This finding has two important implications. First, it confirms that MMR is not invariant. Its amplitude and presence depend critically on stimulus discriminability and temporal context. Second, it suggests that even at a pre-attentive or pre-conscious level, temporal regularity strengthens sensory memory representations and facilitates predictive modeling. When the signal is clear enough, predictable timing makes it more salient.

### 3.3. The P300 Reflects Enhanced Processing of Predictable Deviants

The ERP data from the active condition provide converging neurophysiological evidence for the behavioral findings, revealing how temporal predictability shapes neural responses to deviant sounds. In the difficult condition, a significant P300 was observed for all paradigms. Notably, the P300 amplitude for the oddball paradigm was less increased in comparison to other paradigms. This indicates that when frequency differences are subtle and attention is required, the oddball paradigm with its inherent temporal uncertainty elicited less consistent neural response to deviants. The local irregularity, by contrast, not only rescued this response but also produced a significantly larger P300 than the oddball paradigm in both easy and difficult conditions.

In the easy condition, P300 latency was shortest for the local paradigm (250–500 ms window), indicating faster evaluation and categorization of predictable deviants [31]. In the difficult condition, P300 was delayed across all paradigms, reflecting the increased processing demands of subtle frequency discrimination. Critically, however, the local irregularity still elicited the earliest response among the three and was the only paradigm to show a sustained, significant difference from standards across a broad time window (416–676 ms). This latency shift from approximately 250 ms in the easy condition to 416 ms in the difficult condition for bundles demonstrates that temporal predictability accelerates stimulus evaluation even when discrimination is challenging, though it cannot fully overcome the inherent difficulty of the task.

Cross-paradigm comparisons revealed graded effects. In the easy condition, P300 was larger for the local irregularity compared to both the oddball and two-tone frequency discrimination paradigms. In the difficult condition, this pattern persisted: P300 was larger for the local irregularity than for the oddball) and for the local irregularity than for the two-tone frequency discrimination paradigm. Notably, the two-tone frequency discrimination paradigm also showed a larger P300 than the oddball paradigm, suggesting that even the modest temporal structure of paired sounds (500 ms ISI between tones within a pair) confers some neural advantage over fully unpredictable oddball presentation.

These findings align with predictive coding accounts. P300 is thought to reflect attention-driven context updating when a deviant stimulus is detected and categorized [20]. Its amplitude is proportional to the attentional resources allocated, and its latency indexes stimulus evaluation time [31]. The more precise the temporal prediction, knowing when the deviant will occur, the larger and earlier the prediction error signal when that prediction is violated. Temporal predictability does not simply make deviants easier to detect; it fundamentally alters how the brain processes them, enhancing the neural salience of deviant events and accelerating their categorization. This neural enhancement provides the substrate for the behavioral advantages observed in hit rate and perceptual sensitivity, particularly when discrimination is most challenging.

### 3.4. Dissociation Between Objective Performance and Subjective Awareness

Perhaps the most intriguing finding concerns metacognitive awareness. In the difficult condition, participants performed significantly better in the local paradigm. The participants detected more deviants and showed higher perceptual sensitivity. Yet they did not rate it as subjectively easier than the two-tone frequency discrimination paradigm. When asked to rank paradigms from easiest to most difficult, participants showed no clear consensus. Only in the easy condition did they explicitly recognize that bundles were easier to discriminate.

This dissociation between objective performance and subjective judgment has been observed in other domains, such as visual perception and memory [37], but rarely in auditory frequency discrimination. Why did participants fail to notice their own advantage?

One possibility is that perceptual awareness requires a certain signal-to-noise ratio. When discrimination is near threshold, participants may experience all trials as difficult and uncertain, regardless of paradigm. Their subjective ratings may reflect this global feeling of difficulty rather than a paradigm-by-paradigm comparison. The absolute ratings support this: even for bundles, mean ease ratings remained below 4 on a 7-point scale in the difficult condition. Participants knew the task was hard, but they did not know when they were performing better.

A second, not mutually exclusive possibility is that the benefit of temporal predictability may operate partly below the level of conscious access, as suggested by the dissociation between objective performance and subjective ratings. However, alternative explanations (e.g., global ratings of difficulty, response bias) cannot be ruled out, and our subjective measures (post-block ratings) are less sensitive than trial-by-trial confidence judgments. The correlation data provide some support for this interpretation but should be viewed as suggestive rather than definitive. While subjective ratings of efficiency correlated significantly with hit rate across all paradigms, the correlation with d’, a measure that accounts for both hits and false alarms, fell just short of significance for the oddball paradigm (*p* = 0.055) and was weakest overall in the most temporally uncertain condition. On the one hand, this pattern may suggest that metacognitive accuracy may be poorer when temporal structure is absent, even when sounds are easily discriminable. On the other hand, this may be interpreted as limited rather than absent metacognitive awareness, and we note that our study was not designed as a formal metacognition experiment (which would require trial-by-trial confidence ratings and receiver operating characteristic analysis of metacognitive efficiency.

### 3.5. Individual Differences: One Size Does Not Fit All

We note that our study was not designed or powered to detect individual difference moderators (N = 36 is insufficient for reliable interaction detection). Still, the substantial inter-individual variability observed in this study challenges the notion of a universal ‘optimal paradigm’ for frequency discrimination. While the local irregularity showed a statistically significant advantage at the group level, this advantage was absent from 42% of participants (15/36), who performed best on either the two-tone or oddball paradigm.

Individual differences in auditory processing are well documented. Firstly, musicians, for example, often excel at relative pitch comparisons and may benefit more from two-tone paradigms that require direct comparison of two tones [38]. Secondly, individuals with high working memory capacity [39] may perform well in the oddball paradigms, which require maintaining a stable standard representation over time and comparing each incoming sound to that trace. Conversely, individuals with strong rhythmic or temporal processing abilities may disproportionately benefit from bundle structures that leverage predictable timing. Practically, these findings suggest that auditory assessment batteries should include multiple paradigms rather than relying on any single ‘gold standard,’ as different individuals may show different patterns of strengths and weaknesses.

Importantly, the presence of individual differences strengthens our central claim: the bundle advantage is not universal, and future auditory assessments should consider multiple paradigms to capture the full range of listener abilities.

### 3.6. Limitations and Future Directions

Firstly, while our sample size (N = 36) was adequate for detecting large effects, future studies with larger samples (N > 100) should systematically measure cognitive and perceptual traits to identify predictors of who benefits most from temporal predictability. Moreover, with only 30 deviant trials per condition, we may have been underpowered to detect small MMN effects. Future studies should increase deviant trial counts to 60–100 per condition.

Secondly, we used fixed frequency differences (5–15 Hz for difficult; 40–80 Hz for easy) and a fixed within-bundle ISI (100 ms). It is unknown whether the bundle advantage generalizes to other frequency ranges, different ISI durations, or different numbers of standards per bundle (e.g., three vs. five). Parametric manipulation of these variables in future studies could map the boundary conditions of the effect.

Thirdly, the fixed order of difficulty conditions (difficult always preceding easy) was implemented to minimize carryover effects from easy to difficult blocks, as experiencing easy discrimination first might reduce motivation or induce strategic changes that could artificially impair performance on subsequent difficult blocks. However, we acknowledge that this design choice introduces alternative confounds. First, participants may have experienced learning effects across blocks, potentially improving performance on easy blocks not due to stimulus characteristics but due to increased familiarity with the task structure. Second, fatigue could have accumulated, though the easy blocks being shorter and less demanding may have mitigated this concern. Third, order effects cannot be completely disentangled from difficulty effects. We note that our critical finding—the local irregularity advantage in the difficult condition—emerged despite the difficult condition being presented first, when participants were least practiced. This pattern argues against a simple learning account, as any learning benefit would have favored later-presented conditions, yet the local irregularity advantage was observed in the first set of blocks. Nevertheless, future studies should counterbalance difficulty in order to definitively rule out order effects.

Fourthly, we observed substantial inter-individual variability in which paradigm yielded the best performance. Future studies should systematically assess cognitive traits such as working memory capacity, attention, and rhythmic ability to identify predictors of who benefits most from temporal predictability.

Fifthly, while we measured MMN/MMR and P300, these are coarse indices of predictive processing. Oscillatory dynamics, particularly theta-band (4–8 Hz) phase entrainment, are thought to underlie temporal expectation [40]. It is plausible that local irregularity entrains neural oscillations more effectively than oddball or two-tone frequency discrimination paradigms, leading to enhanced sensory gain at predicted time points. Future studies may analyze inter-trial phase coherence and time-frequency power to test this hypothesis.

Sixthly, we acknowledge that the ERP components examined, eMMN, MMR, and P300, may exhibit temporal overlap, particularly in the passive condition, where the negative component (140–264 ms) could reflect contributions from both pre-attentive and attention-capture processes. While cluster-based permutation tests help isolate significant differences, the functional interpretation of these components should be made with caution, given the potential for overlap.

Seventhly, our passive condition is better characterized as an instructionally ignored condition rather than a truly pre-attentive state [19]. Some participants may have intermittently attended to the sounds despite instructions to ignore them. Thus, our passive state may be better characterized as an instructionally ignored condition rather than a truly pre-attentive state. This may explain why we observed MMR (which reflects involuntary attention capture) rather than pure eMMN. Future studies could use a more rigorous distractor task (e.g., a visual continuous performance task with behavioral responses) to more effectively divert attention or make frequency differences less vivid.

Eighthly, we acknowledge that both ceiling effects in the easy condition and floor effects in the difficult condition (including eight participants who failed to detect any deviants in some paradigms) limit the interpretability of our results. The difficult condition’s low hit rates (30–54% across paradigms) indicate that for many participants, the 5–15 Hz deviation was near or below perceptual threshold under the demanding task conditions. Albeit this allowed us to observe paradigm differences that emerge specifically when discrimination was challenging. However, it means we cannot determine whether temporal predictability would benefit intermediate-difficulty discrimination (e.g., 20–30 Hz deviations) that avoid both floor and ceiling effects. Future studies should include a wider range of deviation sizes (e.g., 5, 15, 25, and 40 Hz) to characterize the full psychometric function.

Finally, it is unknown how the ability to utilize temporal predictability for frequency discrimination develops in children and declines in older adults. Aging is associated with both spectral and temporal processing deficits. Temporal cueing might serve as a compensatory strategy for older listeners, partially offsetting age-related declines in frequency selectivity. Longitudinal or cross-sectional studies are needed to address this question. Understanding the basis of these age-related changes could lead to the development of cognitive training protocols designed to optimize temporal expectation.

## 4. Materials and Methods

### 4.1. Participants

Thirty-six participants (24.36 ± 6.47; age range from 19 to 44 years old; 10 males) without formal musical education (more than two years in musical school) took part in our study. Eligibility criteria for the participants included being aged 18–45, with no history of hearing impairment. All participants were instructed to refrain from alcohol for 24 h and from caffeine (i.e., tea and coffee) for 1–2 h before the laboratory visit. The study was carried out using the equipment of the Research Resource Center of IHNA and NPh RAS for functional brain mapping.

### 4.2. Stimuli and Paradigms

#### 4.2.1. Stimuli

**Figure 4:**
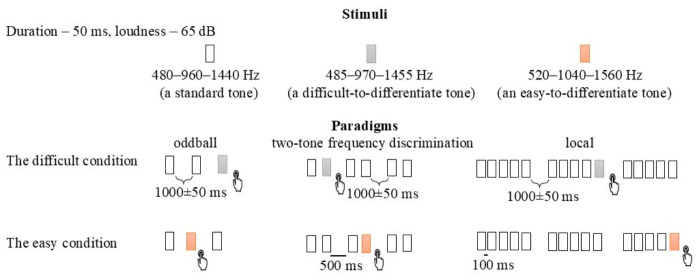
Schematic illustration of the three experimental paradigms.

We used three types of sound stimuli with either a small or a big difference in the frequency filling (Figure 4). Standard was a harmonic tone composed of three sinusoidal partials of 480–960–1440 Hz. The deviant tone differed from a standard tone in frequency, i.e., it was either 485–970–1455 Hz or 520–1040–1560 Hz, respectively. In all three sounds, the intensity of the second and third partials was lower than the first partial by 3 and 6 dB. The duration of each sound was 50 ms (including 5 ms rise and fall times). The stimuli were presented via speakers at 65 dB with equal phase and intensity at both ears. Standards appeared with 80% frequency, and deviants were presented with an equal frequency of 20%. Altogether 150 standard and 30 deviant sounds were presented in each paradigm and block, where the first ten trials were always standard. In total, the whole experiment lasted 2–2.5 h.

#### 4.2.2. Paradigms

We investigated whether the grouping of closely spaced frequencies into rapid sequences improves discrimination compared to isolated or paired presentations (Figure 4, Supplementary S1). The study participants were presented with three modified versions of the paradigms in a pseudo-random order: oddball [4], 2-tone frequency discrimination [6], and local irregularity of the local/global paradigm [7], where they were presented with either single sounds, pairs of sounds, or bundles of sounds, respectively.

In the oddball paradigm, a deviant sound sometimes appeared among the standard sounds. (a a B a a a). SOA was 1000 ms with 50 ms jitter. The sequence lasted for 3 min.

In the 2-tone frequency discrimination paradigm, pairs of sounds were presented (aa aa aB aa aa aa). ISI between stimuli was 500 ms, and SOA between pairs of sounds was 1000 ± 50 ms. The sequence lasted for 4 min.

In the local/global paradigm, sequences consisting of only one type of irregularity were presented: local. The sequence consisted of two types of bundles: one bundle consisted of five identical sounds and standards (aaaaaa), and the second bundle consisted of four identical sounds, standards, and a fifth different, deviant sound (aaaaaB) (aaaaaa aaaaaa aaaaaaB aaaaaa aaaaaa aaaaaa). SOA between bundles was 1000 ± 50 ms. ISI between the sounds within a bundle was 100 ms. The sequence lasted for 5 min.

The order of paradigms was assigned randomly between the participants but not the order of blocks. The stimuli were always presented in a difficult-to-distinguish order followed by an easy-to-distinguish order (Figure 4, Supplementary S1). The order of difficulty conditions was fixed (difficult always preceding easy) for one primary reason, i.e., to escape bias, especially in the first passive state. Not knowing how the sounds should be organized, only those participants who truly heard the difference between sounds reported on that difference. Secondly, they did not want to pay attention to the sounds. However, we acknowledge the limitations of this design (see Limitations).

#### 4.2.3. Experimental Conditions

There were two states: passive and active. In the passive state, the participants were instructed to watch a muted nature documentary and to ignore the auditory stimuli. In the active state, the participants were instructed to press a mouse button as quickly as possible when they detected (or thought they detected, if unsure) a deviant stimulus (i.e., a different sound, pair, or bundle).

Presentation of stimuli sequences, synchronization with EEG recording and registration of participants’ responses were done by Presentation Software 22.1 (Neurobehavioral Systems, Inc., Berkeley, CA, USA).

We analyzed ERPs and subjective rating data obtained during passive and behavioral, ERPs and subjective rating data obtained during active listening to sound sequences.

### 4.3. Behavioral Data

#### 4.3.1. Recording

In active paradigms, mouse button presses were recorded. We analyzed reaction time (RT) (time from deviant onset to button press, ms), percent of correct responses (hit) and d-prime sensitivity index (d’), where hit is the percentage of detected target stimuli; d’ = [z(hit rate) − z(false alarm rate)]/2, where Z (p) is the inverse of the cumulative distribution function of the normal distribution. D’ is a measure of an individual’s ability to detect signals from noise or sensitivity derived from signal detection theory that accounts for both hits and false responses [41].

There were two analyses of RT, i.e., with a limitation to 850 ms after the stimulus onset for ERP analysis and without it.

For behavioral analysis, all deviant trials with response times less than 230 ms after the stimulus onset were deleted from the analysis as they could reflect an automatic response. In two-tone frequency and local irregularity, if motor response were registered during the presentation of a standard or deviant pair or bundle, respectively, but before the start of the target second or the fifth sound, it was related to the previous pair or bundle.

For ERP analysis, all deviant trials with response times less than 230 ms after the stimulus onset and any response in the 100 ms window before the stimulus onset were deleted from the analysis. All standard trials with any preceding or following stimuli onset were deleted.

#### 4.3.2. Statistical Analysis

The data were analyzed in RStudio, version 2026.01.1+403 with the tidyverse, rstatix, ggpubr, and ggsignif packages (Posit Software version 2026.01.1+403, PBC, Boston, MA, USA). Since the data were nonparametric (Shapiro–Wilk test, *p* < 0.05), all data were analyzed with Wilcoxon signed-rank tests with Holm adjustment, conducted only when the Friedman ANOVA was significant. Paired Wilcoxon signed-rank test with Holm correction for multiple comparisons was used to compare two conditions (easy vs. difficult). The significance level was set at α = 0.05 for all tests. All tests were two-tailed.

### 4.4. EEG Data

#### 4.4.1. Recording

Continuous EEG data were recorded from 32 scalp sites (actiCAP snap, Easycap GmbH, Herrsching, Germany), where 32 AgCl electrodes were placed according to an extended international 10–20 system. TP9 served as a reference channel for the monopolar design of EEG recordings. Impedance was kept below 15 kΩ (32-channel actiCHamp Plus, Herrsching, Germany). A bandpass filter was set from 0.05 to 70 Hz, and the sampling rate was 500 Hz.

#### 4.4.2. Preprocessing

EEG data were analyzed in BrainVision Analyzer 2.2 (Brain Products GmbH, Gilching, Germany). First, EEG noisy channels were interpolated, and the EEG signals were referenced to an average reference. A high-pass filter of 0.5 Hz (slope—8 dB/octave), a low-pass filter at 30 Hz (slope—8 dB/octave), and a notch filter at 50 Hz were applied. To remove eye-movement and blink-related EEG artifacts, independent component analysis was conducted. Additionally, continuous EEG was visually inspected and manually cleared of periods with muscle and motion artifacts.

To analyze ERPs in response to stimuli, epochs were segmented from −100 ms to 850 ms after the onset of the last stimulus (one sound, the second sound in a pair, and the fifth sound in a bundle). We used a baseline correction of −100 ms to 0 ms window before the stimulus onset. Additionally, we performed an automatic artifact rejection of EEG epochs. We defined the following criteria for artifact rejection: a voltage step of more than 100 µV/ms, a voltage difference of less than −100 µV or more than 100 µV, and a maximum voltage difference of more than 100 µV within 100 ms intervals. Trial counts after artifact rejection for passive condition: difficult (oddball: standards = 98.25 ± 2.06 (range: 92–100%); deviants = 99.39 ± 0.77 (range: 98–100%); two-tone: standards = 97.8 ± 3.47 (range: 82–100%); deviants = 99.36 ± 0.83 (range: 97–100%); local irregularity: standards = 98.22 ± 2.19 (range: 91–100%); deviants = 99.42 ± 0.73 (range: 98–100%)); easy (oddball: standards = 98.42 ± 2.21 (range: 91–100%); deviants = 99.44 ± 0.73 (range: 98–100%); two-tone: standards = 99.11 ± 1.28 (range: 93–100%); deviants = 99.44 ± 0.73 (range: 98–100%); local irregularity: standards = 98.47 ± 2.04 (range: 92–100%); deviants = 99.19 ± 1.01 (range: 96–100%)). Minimum trial counts exceeded the recommended lower bound of 15–20 trials for reliable ERP estimation.

In the active condition beside noisy trials, trials with false responses or enlarged responses that got into baseline window (−100 0) (for standards) and no responses or responses below 230 ms (for deviants) were excluded from the analysis: difficult (oddball: standards = 87.16 ± 9.74 (range: 57–100%); deviants = 30.67 ± 21.2 (range: 7–80%); two-tone: standards = 89.14 ± 9.90 (range: 60–100%); deviants = 37.02 ± 30.07 (range: 3–97%); local irregularity: standards = 91.82 ± 8.39 (range: 66–100%); deviants = 53.79 ± 31.93 (range: 3–98%)); easy (oddball: standards = 95.87 ± 5.45 (range: 73–100%); deviants = 93.63 ± 9.61 (range: 53–100%); two-tone: standards = 94.03 ± 8.18 (range: 70–100%); deviants = 91.4 ± 11.8 (range: 58–100%); local irregularity: standards = 94.67 ± 6.98 (range: 74–100%); deviants = 92.99 ± 10.11 (range: 53–100%)).

Remaining trials were averaged separately for each stimulus type (standard, deviant), paradigm, condition, and participant and exported for further statistical analysis with “*.ep*” extension.

#### 4.4.3. Statistical Analysis

Statistical comparison of the averaged ERPs’ mean amplitude was performed using FieldTrip Toolbox [42], which is an open-source MATLAB package for EEG/ERP analysis (FieldTrip, MATLAB 2018b, The MathWorks, Inc., Natick, MA, USA). Between-group and within-group differences in ERPs were tested by cluster permutation tests corrected for multiple comparisons. For cluster analysis, the following criteria were chosen: Monte Carlo method, 2-millisecond step, 500 permutations, alpha level for the two-sided permutation *t* tests = 0.025, and corrected cluster-forming threshold = 0.025 on a minimum of 3 neighboring electrodes. For the passive condition, the difference in amplitude in response to the stimuli was analyzed in a 90–300 ms time window for MMN at F3, Fz, F4, FC2, FC1, C3, Cz, and C4 electrodes. For the active condition, the difference in amplitude in response to the stimuli was analyzed in a 250–850 ms window for P300 at CP6, CP2, CP1, CP5, P7, P3, Pz, P4, P8, O1, Oz, and O2 electrodes. The following windows were chosen based on the previous studies (to detect either eMMN or MMR: Refs. [10,18]; for P300 [31]) and adjusted according to the averaged ERPs in the current study and to account for potential latency delays associated with increased perceptual difficulty in processing close-in-frequency sounds.

### 4.5. Self-Report

#### 4.5.1. Questionnaires

In both states (passive and active), we asked participants to evaluate whether it was easy to differentiate sounds (on a scale from 1 to 7, where 1 means no difference between sounds, and 7 means I heard the difference vividly) (see Supplementary S2). Additionally, in the active state, the participants were asked to say how well they performed the task (on a scale from 1 to 7, where 1 means performed very badly, and 7 means performed very well). Some of the questions were adapted from the work by Türker and colleagues [43].

#### 4.5.2. Statistical Analysis

The data were analyzed in RStudio, version 2026.01.1+403 with the tidyverse, rstatix, ggpubr, and ggsignif packages (Posit Software version 2026.01.1+403, PBC, Boston, MA, USA). Since the ease and efficiency ratings were measured on an ordinal scale (1–7), non-parametric tests were employed. Specifically, the Friedman test was employed to assess task differences within each condition, followed by post hoc pairwise comparisons using the Wilcoxon test with Holm adjustment. Paired Wilcoxon signed-rank test with Holm correction for multiple comparisons was used to compare two conditions. The significance level was set at α = 0.05 for all tests. All tests were two-tailed.

Spearman’s rank correlations were computed to assess the relationship between subjective efficiency ratings (1–7 scale) and objective performance measures (hit percentage and d’ sensitivity index), separately for each condition and task.

## 5. Conclusions

This study demonstrates that temporal organization can shape auditory frequency discrimination, particularly under challenging listening conditions. The primary finding of this study is that the local irregularity paradigm, which combines temporal predictability, repeated standard presentation, and short ISI, yielded superior frequency discrimination performance compared to oddball and two-tone frequency discrimination paradigms, but only when discrimination was difficult and attention was active. Under passive listening, no reliable change-detection response was observed for difficult stimuli, suggesting that pre-attentive mechanisms may be insufficient for near-threshold frequency differences under these parameters.

Crucially, these group-level effects masked substantial individual variability. Only 21 of 36 participants showed the ‘bundle advantage,’ while 9 performed best with pairs and 6 with single sounds. This heterogeneity suggests that the optimal paradigm for frequency discrimination depends on individual profiles, a finding with implications for both basic research (paradigm selection should consider individual differences) and clinical applications (temporal cueing may benefit some but not all listeners).

The dissociation between objective performance and subjective ratings was partial and inconsistent, with participants showing some metacognitive insight (significant correlations between subjective efficiency and d’) but failing to explicitly recognize the bundle advantage over pairs. This suggests that the benefits of temporal structure operate partly outside conscious awareness, though formal metacognitive experiments are needed to confirm this interpretation.

These findings may advance our understanding of auditory scene analysis, predictive coding, and attention-perception interactions. They also offer practical guidance for the design of auditory displays and suggest that clinical assessments of auditory function should consider temporal structure as a critical variable, not a nuisance factor.

## Figures and Tables

**Table 1:** Subjective rating of ease to differentiate sounds across paradigms and difficulty levels (the passive state).

Condition/Paradigm	1	2	5	1 vs. 2	1 vs. 5	2 vs. 5
Difficult	2.89 ± 2.28	3.08 ± 2.32	3.56 ± 2.35	0.536	0.507	0.536
Easy	5.75 ± 1.59	5.89 ± 1.60	6.19 ± 1.21	0.235	**0.021**	0.146

Note. 1—oddball paradigm; 2—2-tone frequency discrimination paradigm; 5—local irregularity of the local/global paradigm. Values are presented as mean ± SD. *p*-values are shown for pairwise comparisons between paradigms (1 vs. 2, 1 vs. 5, 2 vs. 5). The statistically significant differences (corrected *p* < 0.05) are highlighted in bold.

**Table 2:** Behavioral performance across paradigms and difficulty levels in 36 participants.

	The Difficult Condition						The Easy Condition					
	*1*	*2*	5	*1* vs. *2*	*1* vs. *5*	*2* vs. *5*	*1*	*2*	5	*1* vs. *2*	*1* vs. *5*	*2* vs. *5*
Hit	30.35 ± 22.43	37.50 ± 31.56	53.06 ± 34.21	**0.04**	**<0.001**	**0.001**	95.79 ± 7.82	96.67 ± 6.85	96.85 ± 6.11	0.196	0.268	1
RT of hits (ms)	615.43 ± 130.55	663.94 ± 156.70	627.42 ± 154.55	0.624	1	0.716	465.94 ± 68.26	472.51 ± 83.68	440.64 ± 80.20	0.529	**0.014**	**0.003**
d’	0.28 ± 1.13	0.58 ± 1.67	1.42 ± 1.73	0.486	**<0.001**	**0.004**	4.22 ± 0.59	4.28 ± 0.58	4.22 ± 0.66	0.942	0.942	0.942

Note. 1—oddball paradigm; 2—two-tone frequency discrimination paradigm; 5—local irregularity; RT—reaction time; ms—millisecond; d’ = sensitivity index. Values are presented as mean ± SD. *p*-values are shown for pairwise comparisons between paradigms (1 vs. 2, 1 vs. 5, 2 vs. 5). The statistically significant differences (*p*-corrected < 0.05) are highlighted in bold.

**Table 3:** Behavioral performance across paradigms and difficulty levels in 31 participants.

	The Difficult Condition						The Easy Condition					
	*1*	*2*	5	*1* vs. *2*	*1* vs. *5*	*2* vs. *5*	*1*	*2*	5	*1* vs. *2*	*1* vs. *5*	*2* vs. *5*
Hits	30.67 ± 21.20	37.02 ± 30.07	53.79 ± 312.93	0.242	**<0.001**	**<0.001**	93.63 ± 9.61	91.4 ± 11.8	92.99 ± 10.12	1	1	1
RT of hits (ms)	542.21 ± 100.1	577.6 ± 78.31	534.85 ± 88.65	**0.022**	0.568	**0.021**	453.1 ± 54.02	456.404 ± 74.24	430.1 ± 73.52	0.839	0.068	**0.024**

Note. 1—oddball paradigm; 2—two-tone frequency discrimination paradigm; 5—local irregularity. *p*-values are shown for pairwise comparisons between paradigms (1 vs. 2, 1 vs. 5, 2 vs. 5). The statistically significant differences (*p*-corrected < 0.05) are highlighted in bold.

**Table 4:** Subjective rating on the ease to differentiate sounds across paradigms and difficulty levels.

	The Difficult Condition						The Easy Condition					
	*1*	*2*	5	*1* vs. *2*	*1* vs. *5*	*2* vs. *5*	*1*	*2*	5	*1* vs. *2*	*1* vs. *5*	*2* vs. *5*
Subjective ease	2.00 ± 1.01	2.75 ± 1.65	3.19 ± 1.70	**0.004**	**<0.001**	0.085	6.58 ± 0.55	6.58 ± 0.73	6.75 ± 0.60	1	0.19	0.06
Subjective efficiency	1.94 ± 0.98	2.67 ± 1.60	3.28 ± 1.63	**0.002**	**<0.001**	**0.032**	6.14 ± 0.76	6.22 ± 1.10	6.39 ± 0.96	0.438	0.092	0.256

Note. 1—oddball paradigm; 2—two-tone frequency discrimination paradigm; 5—local irregularity. The statistically significant differences (corrected *p* < 0.05) are highlighted in bold.

**Table 5:** Subjective ranking of performance ease in 36 participants.

	1 2 5	1 5 2	2 1 5	2 5 1	5 1 2	5 2 1	1 2=5	2 1=5	5 1=2	5=2 1	No Difference
% in the difficult condition	8.3	5.6	8.3	19.4	27.8	25	0	0	5.6	0	0
% in the easy condition	8.3	11.1	5.6	5.6	13.9	19.4	5.6	0	2.8	8.3	19.4

Note. 1—oddball paradigm; 2—two-tone frequency discrimination paradigm; 5—local irregularity.

**Table 6:** Relationship between subjective awareness and objective performance.

d’					
		Spearman R	*p*	Spearman R	*p*
The difficult condition	1	0.52	**0.001**	0.32	0.055
	2	0.63	**<0.001**	0.58	**<0.001**
	5	0.65	**<0.001**	0.66	**<0.001**
The easy condition	1	0.41	**0.012**	0.24	0.162
	2	0.38	**0.021**	0.35	**0.037**
	5	0.49	**0.003**	0.34	**0.045**

Note. 1—oddball paradigm; 2—two-tone frequency discrimination paradigm; 5—local irregularity. The statistically significant differences (*p* < 0.05) are highlighted in bold.

## Data Availability

The datasets presented in this article are not readily available due to the fact that they are part of an ongoing study. Requests to access the datasets should be directed to kliaukovich@ihna.ru.

## Supplementary Materials

The following supporting information can be downloaded at: https://www.mdpi.com/article/10.3390/brainsci16050439/s1, Supplementary S1: Protocol. Supplementary S2: Self-report.

## Abbreviations

The following abbreviations are used in this manuscript:

IHNA & NPh RAS	The Institute of Higher Nervous Activity of the Russian Academy of Sciences
EEG	Electroencephalography
ERP	Event-related potential
MMN	Mismatch negativity
SOA	Stimulus onset asynchrony
ISI	Inter-stimulus interval
RT	Reaction time
ms	Millisecond

## Appendix A

**Table A1:** Behavioral results and subjective rating of ease to differentiate sounds across paradigms and difficulty levels (the passive and active states). Comparison of three tasks (oddball vs. two-tone vs. local).

Condition/Paradigm		1 vs. 2				1 vs. 5				2 vs. 5			
		N	W	*p*	*p*-Corr	N	W	*p*	*p*-Corr	N	W	*p*	*p*-Corr
Subjective ease (passive state)	Difficult	36	18	0.353	0.536	36	84	1.169	0.507	36	102	0.268	0.536
	Easy	36	13	0.235	0.235	36	0	0.007	**0.021**	36	2	0.073	0.146
Hits	Difficult	36	154	0.04	**0.04**	36	50	<0.001	**<0.001**	36	94	<0.001	**<0.001**
	Easy	36	43	0.204	0.612	36	68	0.275	0.612	36	68	1	1
RT of hits (ms)	Difficult	33	209	0.208	0.624	34	281	1	1	35	333	0.358	0.716
	Easy	36	292	0.529	0.529	36	501	0.007	**0.014**	36	532	0.001	**0.003**
d’	Difficult	36	272	0.486	0.486	36	81	<0.001	**<0.001**	36	128	0.002	**0.004**
	Easy	36	70	0.333	0.999	36	102	0.94	0.999	36	113	0.481	0.999
Subjective ease	Difficult	36	35	0.002	**0.004**	36	17	<0.001	**<0.001**	36	74	0.085	0.085
	Easy	36	23	1	1	36	9	0.095	0.19	36	0	0.02	0.06
Subjective efficiency	Difficult	36	40	0.001	**0.002**	36	11	<0.001	**<0.001**	36	54	0.32	**0.032**
	Easy	36	50	0.539	0.539	36	60	0.064	0.192	36	43	0.141	0.282
Hits	Difficult	31	119	0.242	0.242	31	65	<0.001	**<0.001**	31	49	<0.001	**<0.001**
	Easy	31	134	0.531	1	31	153	0.67	1	31	76	0.456	1
RT of hits (ms)	Difficult	31	120	0.011	**0.022**	31	278	0.568	0.568	31	384	0.007	**0.021**
	Easy	31	237	0.839	0.839	31	356	0.034	0.068	31	381	0.008	**0.024**

Note. 1—oddball paradigm; 2—two-tone frequency discrimination paradigm; 5—local irregularity. *p*-values are shown for pairwise comparisons between paradigms (1 vs. 2, 1 vs. 5, 2 vs. 5) and between difficulty levels for each paradigm. The statistically significant differences (*p*-corrected (corr) < 0.05) are highlighted in bold.

**Table A2:** Behavioral results and subjective rating of ease to differentiate sounds across paradigms and difficulty levels (the passive and active states). Comparison of two conditions (difficult vs. easy).

Condition/Paradigm	1				2				5			
	N	W	*p*	*p*-Corr	N	W	*p*	*p*-Corr	N	W	*p*	*p*-Corr
Subjective ease (passive state)	36	24	<0.001	**<0.001**	36	45.5	<0.001	**<0.001**	36	29	<0.001	**<0.001**
Hits	36	0	<0.001	**<0.001**	36	0	<0.001	**<0.001**	36	7.5	<0.001	**<0.001**
RT of hits (ms)	34	572	<0.001	**<0.001**	35	630	<0.001	**<0.001**	36	666	<0.001	**<0.001**
d’	36	0	<0.001	**<0.001**	36	0	<0.001	**<0.001**	36	9	<0.001	**<0.001**
Subjective ease	36	0	<0.001	**<0.001**	36	0	<0.001	**<0.001**	36	0	<0.001	**<0.001**
Subjective efficiency	36	0	<0.001	**<0.001**	36	0	<0.001	**<0.001**	36	5	<0.001	**<0.001**
Hits	31	0	<0.001	**<0.001**	31	0	<0.001	**<0.001**	31	11.5	<0.001	**<0.001**
RT of hits (ms)	31	441	<0.001	**<0.001**	31	476	<0.001	**<0.001**	31	469	<0.001	**<0.001**

Note. 1—oddball paradigm; 2—two-tone frequency discrimination paradigm; 5—local irregularity. *p*-values are shown for pairwise comparisons between paradigms (1 vs. 2, 1 vs. 5, 2 vs. 5) and between difficulty levels for each paradigm. The statistically significant differences (*p*-corrected (corr) < 0.05) are highlighted in bold.
