# Supplementary material for: Does Sound Timing Organization Matter? How Time Interval Influences the Perception of Closely Spaced Frequencies
Source: Brain Sci. 2026 Apr 22;16(5):439. doi: 10.3390/brainsci16050439 (PMC13204789; doi:10.3390/brainsci16050439)
Supplement: Supplementary file 1 [file brainsci-16-00439-s001.zip › brainsci-4220864-supplementary.pdf]

brain sciences

MDPI

Supplementary S1. Protocol

Date________ID ___________________ Variant ________________

Time	Test	Comments
	**Informed consent**	
	**Audiometry**	
	**Resting state**	
	Passive_	
	Passive_	
	Passive_	
	**Questionnaire**	
	Learning	
	Active_	
	**Questionnaire**	
	Learning	
	Active_	
	**Questionnaire**	
	Learning	
	Active_	
	**Questionnaire**	
	Arrange the sequences in order from the easiest to distinguish to the most difficult:	
	Passive_ _2	
	Passive_ _2	
	Passive_ _2	
	**Questionnaire**	
	Passive_easy_	
	Passive_easy_	
	Passive_easy_	
	**Questionnaire**	
	Active_easy_	
	**Questionnaire**	
	Active_easy_	
	**Questionnaire**	
	Active_easy_	
	**Questionnaire**	
	Arrange the sequences in order from the easiest to distinguish to the most difficult:	
	**Resting state**	

Supplementary S2. Self-report

ID ____________________ Variant ________________

1. Questionnaire for the passive listening block:

1. How focused were you during the task (from 1 to 7, where 1 – mind-wandering / listening to sounds, 7 – focused on the task of watching the video)?

   1 2 3 4 5 6 7

   I was mostly:

   A) watching the video

   B) mind-wandering

   C) listening carefully to the sound sequences

   D) other (please describe)

2. How tired were you during the task (from 1 to 7, where 1 – not tired, 7 – very tired)?

   1 2 3 4 5 6 7

3. Did you notice a pattern for each of the sequences?

   For the 1st sequence:

   For the 2nd sequence:

   For the 3rd sequence:

4. If yes, how easy was it for you to distinguish the sounds from each other (from 1 to 7, where 1 – did not distinguish at all, 7 – easily distinguished)?

   ( ) Score for the 1st sequence: 1 2 3 4 5 6 7

   ( ) Score for the 2nd sequence: 1 2 3 4 5 6 7

   ( ) Score for the 3rd sequence: 1 2 3 4 5 6 7

https://doi.org/10.3390/xxxxx

ID ____________________ Variant ________________

2. Questionnaire for the active listening block (oddball paradigm):

1. How focused were you during the task (from 1 to 7, where 1 – mind-wandering, 7 – focused on the task of distinguishing sounds)?

   1 2 3 4 5 6 7

   I was mostly:

   A) mind-wandering

   B) listening carefully to the sound sequences

   C) other (please describe)

2. How tired were you during the task, where 1 – not tired, and 7 – very tired?

   1 2 3 4 5 6 7

3. How easy was it for you to distinguish the sounds from each other (from 1 to 7, where 1 – did not distinguish at all, 7 – easily distinguished)?

   1 2 3 4 5 6 7

4. How successfully did you perform the task (from 1 to 7, where 1 – made mistakes all the time, 7 – no mistakes)?

   1 2 3 4 5 6 7

https://doi.org/10.3390/xxxxx

ID ____________________ Variant ________________

2. Questionnaire for the active listening block (2-tone frequency discrimination paradigm):

1. How focused were you during the task (from 1 to 7, where 1 – mind-wandering, 7 – focused on the task of distinguishing sounds)?

   1 2 3 4 5 6 7

   I was mostly:

   A) mind-wandering

   B) listening carefully to the sound sequences

   C) other (please describe)

2. How tired were you during the task, where 1 – not tired, and 7 – very tired?

   1 2 3 4 5 6 7

3. How easy was it for you to distinguish the sounds from each other (from 1 to 7, where 1 – did not distinguish at all, 7 – easily distinguished)?

   1 2 3 4 5 6 7

4. How successfully did you perform the task (from 1 to 7, where 1 – made mistakes all the time, 7 – no mistakes)?

   1 2 3 4 5 6 7

ID ____________________ Variant ________________

2. Questionnaire for the active listening block (local):

1. How focused were you during the task (from 1 to 7, where 1 – mind-wandering, 7 – focused on the task of distinguishing sounds)?

   1 2 3 4 5 6 7

   I was mostly:

   A) mind-wandering

   B) listening carefully to the sound sequences

   C) other (please describe)

2. How tired were you during the task, where 1 – not tired, and 7 – very tired?

   1 2 3 4 5 6 7

3. How easy was it for you to distinguish the sounds from each other (from 1 to 7, where 1 – did not distinguish at all, 7 – easily distinguished)?

   1 2 3 4 5 6 7

4. How successfully did you perform the task (from 1 to 7, where 1 – made mistakes all the time, 7 – no mistakes)?

   1 2 3 4 5 6 7

ID ____________________ Variant ________________

3. Questionnaire for the passive listening block (difficult-to-differentiate sounds):

1. How focused were you during the task (from 1 to 7, where 1 – mind-wandering / listening to sounds, 7 – focused on the task of watching the video)?

   1 2 3 4 5 6 7

   I was mostly:

   A) mind-wandering

   B) mind-wandering

   C) listening carefully to the sound sequences

   D) other (please describe)

2. How tired were you during the task (from 1 to 7, where 1 – not tired, 7 – very tired)?

   1 2 3 4 5 6 7

3. How easy was it for you to distinguish the sounds from each other (from 1 to 7, where 1 – did not distinguish at all, 7 – easily distinguished)?

   (   ) Score for the 1st sequence: 1 2 3 4 5 6 7

   (   ) Score for the 2nd sequence: 1 2 3 4 5 6 7

   (   ) Score for the 3rd sequence: 1 2 3 4 5 6 7

https://doi.org/10.3390/xxxxx

ID ____________________ Variant ________________

3. Questionnaire for the passive listening block (easy-to-differentiate sounds):

1. How focused were you during the task (from 1 to 7, where 1 – mind-wandering / listening to sounds, 7 – focused on the task of watching the video)?

   1 2 3 4 5 6 7

   I was mostly:

   A) watching the video

   B) mind-wandering

   C) listening carefully to the sound sequences

   D) other (please describe)

2. How tired were you during the task (from 1 to 7, where 1 – not tired, 7 – very tired)?

   1 2 3 4 5 6 7

3. How easy was it for you to distinguish the sounds from each other (from 1 to 7, where 1 – did not distinguish at all, 7 – easily distinguished)?

   ( ) Score for the 1st sequence: 1 2 3 4 5 6 7

   ( ) Score for the 2nd sequence: 1 2 3 4 5 6 7

   ( ) Score for the 3rd sequence: 1 2 3 4 5 6 7

https://doi.org/10.3390/xxxxx

ID ____________________ Variant ________________

4. Questionnaire for the active listening block (oddball paradigm):

1. How focused were you during the task (from 1 to 7, where 1 – mind-wandering, 7 – focused on the task of distinguishing sounds)?

   1 2 3 4 5 6 7

   I was mostly:

   A) mind-wandering

   B) listening carefully to the sound sequences

   C) other (please describe)

2. How tired were you during the task, where 1 – not tired, and 7 – very tired?

   1 2 3 4 5 6 7

3. How easy was it for you to distinguish the sounds from each other (from 1 to 7, where 1 – did not distinguish at all, 7 – easily distinguished)?

   1 2 3 4 5 6 7

4. How successfully did you perform the task (from 1 to 7, where 1 – made mistakes all the time, 7 – no mistakes)?

   1 2 3 4 5 6 7

ID ____________________ Variant ________________

4. Questionnaire for the active listening block (2-tone frequency discrimination paradigm):

1. How focused were you during the task (from 1 to 7, where 1 – mind-wandering, 7 – focused on the task of distinguishing sounds)?

   1 2 3 4 5 6 7

   I was mostly:

   A) mind-wandering

   B) listening carefully to the sound sequences

   C) other (please describe)

2. How tired were you during the task, where 1 – not tired, and 7 – very tired?

   1 2 3 4 5 6 7

3. How easy was it for you to distinguish the sounds from each other (from 1 to 7, where 1 – did not distinguish at all, 7 – easily distinguished)?

   1 2 3 4 5 6 7

4. How successfully did you perform the task (from 1 to 7, where 1 – made mistakes all the time, 7 – no mistakes)?

   1 2 3 4 5 6 7

https://doi.org/10.3390/xxxxx

ID ____________________ Variant ________________

4. Questionnaire for the active listening block (local):

1. How focused were you during the task (from 1 to 7, where 1 – mind-wandering, 7 – focused on the task of distinguishing sounds)?

   1 2 3 4 5 6 7

   I was mostly:

   A) mind-wandering

   B) listening carefully to the sound sequences

   C) other (please describe)

2. How tired were you during the task, where 1 – not tired, and 7 – very tired?

   1 2 3 4 5 6 7

3. How easy was it for you to distinguish the sounds from each other (from 1 to 7, where 1 – did not distinguish at all, 7 – easily distinguished)?

   1 2 3 4 5 6 7

4. How successfully did you perform the task (from 1 to 7, where 1 – made mistakes all the time, 7 – no mistakes)?

   1 2 3 4 5 6 7

https://doi.org/10.3390/xxxxx
